# Supplementary material for: Optimized GENE TARGET framework characterizes barriers to therapeutic development for monogenic neurodevelopmental disorders
Source: Mol Ther Adv. 2026 Oct 17;34(4):201871. doi: 10.1016/j.omta.2026.201871 (PMC13769315; doi:10.1016/j.omta.2026.201871)
Supplement: Supplementary file 1 [file mmc1.pdf]

OMTA, Volume *34*

Supplemental information

Optimized GENE TARGET framework characterizes barriers to therapeutic development for monogenic neurodevelopmental disorders

Raymond Belanger Deloge, Abigail Sveden, Zoë Frazier, Hailey Osika, Charlotte Byrne, Jillian O'Toole, Muhammed Bayraktutan, Kira A. Dies, Stephanie J. Brewster, Pilar Trelles, Morgan E. Ryan, Bo Zhang, Lisa Prock, Jed Hubbs, Elizabeth D. Buttermore, Wendy K. Chung, Audrey Thurm, Mustafa Sahin, and Maya Chopra

Table S1. Inter-rater agreement using ICC and Gwet's AC2

Category	Raters 1 to 3 (95% CI)	Raters 1 and 2 (95% CI)	Raters 1 and 3 (95% CI)	Raters 2 and 3 (95% CI)	Raters 4 and 5 (95% CI)
**Total Score***	0.67 (0.43, 0.84)	0.75 (0.47, 0.89)	0.51 (0.12, 0.77)	0.70 (0.30, 0.88)	0.32 (-0.15, 0.66)
**Criteria**					
G1°	0.86 (0.74,0.98)	0.90 (0.80,1.00)	0.78 (0.57,0.99)	0.77 (0.57,0.98)	0.59 (0.30,0.89)
E1°	0.85 (0.68,1.00)	0.76 (0.48,1.00)	1.00 (1.00, 1.00)	0.76 (0.48,1.00)	0.73 (0.54,0.92)
N°	0.95 (0.87,1.00)	0.96 (0.90,1.00)	0.94 (0.82,1.00)	0.96 (0.89,1.00)	0.89 (0.78,1.00)
E2°	0.43 (0.19,0.67)	0.37 (-0.03,0.77)	0.48 (0.20,0.76)	0.57 (0.33,0.81)	0.83 (0.68,0.98)
T1°	0.82 (0.72,0.91)	0.79 (0.68,0.91)	0.90 (0.79,1.00)	0.75 (0.60,0.90)	0.51 (0.23,0.79)
A°	0.91 (0.80,1.00)	0.93 (0.78,1.00)	0.87 (0.70,1.00)	0.94 (0.86,1.00)	0.86 (0.67,1.00)
R°	0.63 (0.50,0.76)	0.66 (0.44,0.88)	0.57 (0.42,0.72)	0.72 (0.57,0.87)	0.33 (-0.09,0.75)
G2°	0.69 (0.49,0.88)	0.77 (0.61,0.93)	0.67 (0.47,0.87)	0.65 (0.32,0.97)	0.44 (0.02,0.85)
E3°	0.63 (0.48,0.79)	0.67 (0.41,0.93)	-0.19 (-0.67,0.30)	0.63 (0.36,0.90)	0.38 (0.12,0.64)
T2°	0.80 (0.62,0.98)	0.87 (0.73,1.00)	0.56 (0.15,0.96)	0.68 (0.39,0.97)	0.52 (0.10,0.95)
**Domain**					
Clinical°	0.78 (0.67,0.89)	0.69 (0.48,0.89)	0.76 (0.58,0.95)	0.77 (0.66,0.88)	0.52 (0.18,0.86)
Genetic°	0.65 (0.48,0.82)	0.77 (0.61,0.94)	0.52 (0.17,0.86)	0.42 (0.14,0.70)	0.41 (-0.03,0.85)
Pre-Clinical°	0.77 (0.70,0.84)	0.76 (0.63,0.89)	0.77 (0.66,0.88)	0.74 (0.65,0.84)	0.48 (0.15,0.80)
Ethical°	0.64 (0.41,0.88)	0.62 (0.32,0.91)	0.21 (-0.16,0.58)	0.59 (0.21,0.96)	0.21 (-0.20,0.61)

*=Calculated using ICC
o= Calculated using Gwet's AC2

Table S2. Full GENE TARGET Scorecards for all 57 NDDs

Table S3. Criteria and Domain Scores for all 57 NDDs

Table S4. GENE TARGET Score Descriptions

**G**	0 - genetic mechanism is unknown; recommend establishing before proceeding<br>1 - genetic mechanism is unknown, but gene harbors special characteristics that can be exploited<br>2 - multi-gene CNVs account for most cases of the disorder OR conflicting or limited evidence of mechanism<br>3 - conflicting or limited evidence of mechanism, but gene harbors special characteristics that can be exploited<br>4 - loss of function or altered function of a single gene<br>6 - in addition, gene harbors special characteristics that can be exploited
**E**	0 - age of diagnosis or clinical presentation is highly variable<br>1 - diagnosis / clinical presentation in infantile to early childhood period<br>2 - features or markers typically present in prenatal imaging/testing OR clinical presentation of features at birth
**N**	0= No natural history data available; recommend natural history studies before proceeding<br>1= Cross-sectional data available<br>2= Longitudinal data available<br>3= Longitudinal data with standardized neurodevelopmental measures available
**E**	0= No validated endpoints, recommend establishing endpoints before proceeding<br>1= Neurodevelopmental endpoints (ie IQ) available<br>2= Indirect measures/biomarkers associated with neurodevelopmental phenotype available<br>3= Direct endpoints available; how patients feel, function, and survive
**T**	0 - Cell type and/or brain region not defined, no model system<br>1 - Model system under development, cell type and/or brain region not defined<br>2 - Model system available, cell type and/or brain region not defined<br>4 - Cell type and/or brain region and stage of neuronal development known and informed by a validated preclinical model, but targetability limited by properties of brain region<br>6 - Cell type and/or brain region and stage of neuronal development known and informed by a validated preclinical model, and appropriate tools are available which can access that cell type or brain region
**A**	0 - disease-modifying treatments are available and approved by regulators<br>1 - disease-modifying treatments are limited in population, symptomatic domain, or duration of use OR treatment(s) currently under evaluation

	2 - no disease-modifying treatments are available
**R**	0 - reversibility not established and resources are not available; recommend establishing before proceeding<br>1 - reversibility not established, but resources (iPSCs, animal models, organoids, etc) are publicly available and accessible<br>2 - Disorder-relevant preclinical endpoint established in a model, and ready for testing reversibility<br>3 - reversibility established but temporal window unknown<br>4 - reversibility and temporal window for rescue established
**G**	0= Gene tolerance to dosage change is unknown; recommend establishing dosage window before proceeding<br>2= Narrow therapeutic window<br>3= Wide therapeutic window suspected<br>4= Wide therapeutic window confirmed
**E**	0= Ethical principles have not been considered OR are unfavorable<br>3= Favorable risk-benefit ratio OR treatment generalizable across population<br>6= Favorable risk-benefit ratio AND treatment generalizable across population
**T**	0= Patient population not accessible or not engaged<br>2= Engaged population accessible through community organizations<br>4= Engaged population accessible through clinical cohorts or registries
