# Supplementary material for: Optimized GENE TARGET framework characterizes barriers to therapeutic development for monogenic neurodevelopmental disorders
Source: Mol Ther Adv. 2026 Oct 17;34(4):201871. doi: 10.1016/j.omta.2026.201871 (PMC13769315; doi:10.1016/j.omta.2026.201871)
Supplement: Supplementary file 2 [file mmc3.pdf]

Molecular Therapy
Advances
Original Article

Optimized GENE TARGET framework characterizes barriers to therapeutic development for monogenic neurodevelopmental disorders

Raymond Belanger Deloge,[1,2] Abigail Sveden,[1,2] Zoë Frazier,[1,2] Hailey Osika,[1,2] Charlotte Byrne,[3] Jillian O'Toole,[1,2] Muhammed Bayraktutan,[1,2] Kira A. Dies,[1,2] Stephanie J. Brewster,[1,4] Pilar Trelles,[1,5] Morgan E. Ryan,[6] Bo Zhang,[2,6] Lisa Prock,[1,7,8] Jed Hubbs,[1,2,8] Elizabeth D. Buttermore,[1,2,8] Wendy K. Chung,[8,9] Audrey Thurm,[1,5,8] Mustafa Sahin,[1,2,8] and Maya Chopra[1,2,4,8]

[1]Rosamund Stone Zander and Hansjoerg Wyss Translational Neuroscience Center, Boston Children's Hospital, Boston, MA 02115, USA; [2]Department of Neurology, Boston Children's Hospital, Harvard Medical School, Boston, MA, USA; [3]MGH Institute of Health Professions, Boston, MA, USA; [4]Division of Genetics and Genomics, Boston Children's Hospital, Harvard Medical School, Boston, MA, USA; [5]Psychiatry and Behavioral Sciences, Boston Children's Hospital, Boston, MA, USA; [6]Biostatistics and Research Design Center, Institutional Centers for Clinical & Translational Research, Boston Children's Hospital, Harvard Medical School, Boston, MA, USA; [7]Division of Developmental Medicine, Developmental Medicine Center, Boston Children's Hospital, Harvard Medical School, Boston, MA, USA; [8]Harvard Medical School, Boston, MA, USA; [9]Department of Pediatrics, Boston Children's Hospital, Boston, MA, USA

GENE TARGET is a previously published framework designed to quantitatively assess the suitability of a specific monogenic neurodevelopment disorder (NDD) for development of genetic therapies, addressing the need for a systematic and rigorous approach to research prioritization and resource allocation. Here, to keep pace with the rapidly evolving knowledgebase, we refined the framework with more nuanced scoring definitions and added a resource guide to standardize evaluation of each criterion. Interrater agreement was assessed and found to be substantial for most domain/criteria scores among experienced users, primarily genetic counselors with at least two years of experience in translational genomic medicine. Secondary scoring adjudication by experts with clinical/molecular genetics and preclinical modeling expertise is recommended. Cross-disorder analysis of 57 NDD gene-disorder pairs evaluated at the Translational Neuroscience Center was performed, which identified clinical considerations, including establishing endpoints, as the most common translational research gap. Common barriers across disorders also included generating natural history data, establishing an early diagnosis, understanding dosage sensitivity, and demonstrating phenotypic reversibility. We further discuss application of this framework for gene editing and small molecules approaches. Future goals include the validation of GENE TARGET beyond academic research settings and the use of artificial intelligence to aid in preliminary scoring.

INTRODUCTION

Recent advances in genomic sequencing and improved access to genetic testing have increasingly identified monogenic diagnoses in individuals with neurodevelopmental disorders (NDDs), and opened the door to considering multiple strategies for manipulating gene expression or targeting gene products. Therapeutic modalities including gene replacement therapy, antisense oligonucleotides (ASOs), and small molecule intervention have demonstrated promise in treating monogenic disorders.[1–3] Identifying which monogenic conditions should be prioritized for genetic therapies is crucial for academic researchers, drug developers, and patient advocacy groups (PAGs) for the rational and ethical distribution of resources. Given that 95% of rare disorders currently do not have a Food and Drug Administration (FDA)-approved treatment option, there is a need for thoughtful and targeted selection of monogenic conditions for accelerated therapeutic development.[4] Shared and systematic frameworks for evaluating readiness for therapeutic development can facilitate more efficient prioritization, strengthen alignment and collaboration across stakeholders, and ultimately improve outcomes for individuals and families affected with monogenic NDDs. This is particularly important in settings where resources are limited and decisions regarding investment, trial readiness, and therapeutic risk must be made transparently and strategically.

Resources that capture the breadth and depth of scientific and ethical considerations for readiness for drug development are limited, particularly those that are focused on genetic therapies for monogenic NDDs, which are often diagnosed in early childhood. The National Center for Advancing Translational Sciences (NCATS)

Received 30 April 2026; accepted 15 October 2026;
https://doi.org/10.1016/j.omta.2026.201871.

Correspondence: Maya Chopra, Rosamund Stone Zander and Hansjoerg Wyss Translational Neuroscience Center, Boston Children's Hospital, Boston, MA 02115, USA.
E-mail: maya.chopra@childrens.harvard.edu

© 2026 The Author(s).
Published by Elsevier Inc. on behalf of The American Society of Gene and Cell Therapy.
This is an open access article under the CC BY license (http://creativecommons.org/licenses/by/4.0/).

Toolkit for Patient-Focused Therapy Development provides helpful and educational content about the development process from discovery through to post-FDA approval.[5] However, it does not specifically focus on genetic therapy development for brain disorders or disorders with a pediatric onset. The Child Neurology Society's consensus on gene-targeted therapies for pediatric neurological disorders identifies key developmental, diagnostic, and delivery challenges specific to gene therapy readiness in children, highlighting the need for specialized frameworks tailored to pediatric monogenic brain disorders.[6]

In 2022, we published the GENE TARGET framework to facilitate the systematic evaluation of monogenic NDDs for suitability for genetic therapies, with a focus on treatments that target expression of a single gene at a DNA level (principally virus-mediated gene delivery) and at an RNA level (principally expression modulators including ASOs).[7] This tool was initially designed to serve as a shared reference for academic investigators, PAGs, and industry partners working toward improved therapeutic outcomes for monogenic NDDs. The purpose of this tool was 2-fold: first, to identify gaps in the translational pipeline for a specific gene-disorder pair to inform future research efforts, and second, to help researchers compare the readiness of these processes for specific conditions to prioritize those most suitable for therapeutic development.

Scoring considerations for GENE TARGET have been described in detail.[7] Recognizing that a given gene may be implicated in more than one disorder, this framework is designed to be used on a specific gene-disorder pair. Briefly, a rubric is used to score ten criteria that fall into four domains—molecular genetic mechanism, preclinical modeling, clinical trial readiness, and ethical considerations. In the genetic domain, we discussed that the genetic mechanism of a specific disorder (for example loss of function, gain of function) informs the therapeutic approach (for example, gene replacement/upregulation, or knockdown, respectively), considering genomic architecture, isoform specificity, gene dosage windows, and the mutational spectrum. Evaluation for the presence of "special characteristics" (including for example, the presence of a non-productive transcript in a gene with a haploinsufficiency mechanism, which could be targeted with an ASO approach) was also outlined. In the preclinical domain, tissue and temporal specificity, and the availability of a preclinical model system in which reversibility of a disease-relevant phenotype can be demonstrated, was discussed. Clinical factors centered around the typical age of diagnosis (to be considered in the context of the peak expression of the particular gene), the availability of rigorous, longitudinal natural history data and fit-for-purpose clinical trial outcome measures and biomarkers, and whether existing approved therapeutic options were already available. Finally, the ethical considerations were focused on an engaged, accessible patient population desiring of treatments, the risk benefit ratio, and the likelihood that treatment could be generalizable to the entire patient population with that specific genetic disorder, closely tied to a unifying genetic mechanism that was targetable at a DNA or RNA level.

Each of these domains is equally weighted, yielding a maximum gene target suitability (GTS) score of 40. A higher score indicates better therapeutic suitability. With this framework, the suitability of monogenic NDDs for therapeutic development can be quantitatively assessed, and scores can be compared across disorders. Notably, rather than using a rigid numerical cutoff for suitability, GENE TARGET generates a multi-domain profile to identify critical translational roadblocks and guide resource allocation. This approach avoids the masking of actionable bottlenecks—such as natural history gaps—behind a single pass/fail metric. Furthermore, scores for a single disorder may also be tracked longitudinally to monitor translational research progress over time.

At Boston Children's Hospital, the Rosamund Stone Zander and Hansjoerg Wyss Translational Neuroscience Center (TNC) is focused on the acceleration of clinical trial readiness and therapeutic development for rare NDDs. Since publication, we have been using GENE TARGET in the TNC to systematically evaluate requests for core services, collaborative research opportunities, and internal funding priorities. Rather than evaluating disorders on GTS score alone, we consider all criteria to identify research gaps that need to be prioritized for each disorder. In parallel to our Center's experience, researchers and PAGs have independently utilized GENE TARGET. For instance, researchers studying succinic semialdehyde dehydrogenase deficiency (SSADHD) employed the framework to examine the translational landscape and highlight research gaps for this condition.[8] Similarly, the SCN8A Research Consortium referenced GENE TARGET in their research roadmap, emphasizing the critical importance of understanding the gene's dosage sensitivity.[9] GENE TARGET evaluations of *CLCN4*-related disorder, ReNU syndrome, and Kleefstra syndrome have been presented at disorder-specific scientific meetings in strategic planning sessions focused on research roadmaps. In defining the considerations for prenatal gene editing for genetic NDDs, Major et al. evaluated GENE TARGET in tandem with criteria specific to the prenatal setting.[10] The widespread application and recognition of the framework demonstrate its value in addressing the need for a systematic, quantitative approach to research prioritization.

To date, the framework has not undergone systematic validation, nor has interrater agreement been assessed. In light of the limitations identified through use of the initial framework—in particular, the observations that scoring definitions are not sufficiently nuanced and research capabilities in the field have expanded over the last few years—we iteratively refined GENE TARGET. Scoring definitions have been clarified, user recommendations have been defined, and resources and databases from which information should be used for scoring each criterion have been specified. To evaluate scoring consistency across users, interrater agreement was assessed among five users.

Here, we share full scorecards of the gene-disease pairs evaluated in our Center using the updated framework to serve as a resource for researchers, PAGs, and industry partners engaged in the selection

Table 1. Updated GENE TARGET framework

Genetic mechanism is understood and amenable	
0	Genetic mechanism is unknown; recommend establishing before proceeding
1	***Genetic mechanism is unknown but gene harbors special characteristics that can be exploited***
2	Multi-gene CNVs account for most cases of the disorder ***OR conflicting or limited evidence of mechanism***
3	***Conflicting or limited evidence of mechanism, but gene harbors special characteristics that can be exploited***
4	Loss of function or altered function of a single gene
6	In addition, gene harbors special characteristics that can be exploited
Early diagnosis is typical	
0	Age of diagnosis ***or clinical presentation*** is highly variable
1	Diagnosis/***clinical presentation*** in infantile to early childhood period
2	***Features or markers typically present in prenatal imaging/testing OR clinical presentation of features at birth***
Natural history is understood	
0	No natural history data available; recommend natural history studies before proceeding
1	Cross-sectional data available
2	Longitudinal data available
3	Longitudinal data with standardized neurodevelopmental measures available
Endpoints are validated and meaningful	
0	No validated endpoints, recommend establishing endpoints before proceeding
1	Neurodevelopmental endpoints (i.e., IQ) available
2	Indirect measures/biomarkers associated with neurodevelopmental phenotype available
3	Direct endpoints available; how patients feel, function, and survive
Tools deliver to target tissues at the right time	
0	***Cell type and/or brain region not defined, no model system***
1	***Model system under development, cell type and/or brain region not defined***
2	***Model system available, cell type and/or brain region not defined***
4	***Cell type and/or brain region and stage of neuronal development known and informed by a validated preclinical model, but targetability limited by properties of brain region***
6	***Cell type and/or brain region and stage of neuronal development known and informed by a validated preclinical model, and appropriate tools are available which can access that cell type or brain region***
Availability of other safe and effective treatments is limited	
0	Disease-modifying treatments are available and approved by regulators
1	Disease-modifying treatments are limited in population, symptomatic domain, or duration of use ***OR treatment(s) currently under evaluation***
2	No disease-modifying treatments are available
Reversibility has been demonstrated in a model system	
0	Reversibility not established and resources are not available; recommend establishing before proceeding
1	***Reversibility not established, but resources (iPSCs, animal models, organoids, etc.) are publicly available and accessible***
2	***Disorder-relevant preclinical endpoint established in a model, and ready for testing reversibility***
3	Reversibility established but temporal window unknown
4	Reversibility and temporal window for rescue established

(*Continued on next page*)

Table 1. ***Continued***

Gene is tolerant to dosage changes	
0	Gene tolerance to dosage change is unknown; recommend establishing dosage window before proceeding
2	Narrow therapeutic window
3	***Wide therapeutic window suspected***
4	Wide therapeutic window confirmed
Ethical principles have been considered	
0	Ethical principles have not been considered ***OR are unfavorable***
3	Favorable risk-benefit ratio OR treatment generalizable across population
6	Favorable risk-benefit ratio AND treatment generalizable across population
Target population is accessible and engaged	
0	Patient population not accessible or not engaged
2	Engaged population accessible through community organizations
4	Engaged population accessible through clinical cohorts or registries

Bold and italicized text represents new or revised scores and/or score descriptions.

and evaluation of disorders. We also share a comparison of domain and criteria scores across all conditions to highlight the most significant aggregate barriers to therapeutic development across conditions. In the original paper, gene replacement therapy and RNA-based strategies, including the multiple applications of ASO therapies, were extensively discussed. Here, we update considerations for other genetic medicine modalities, specifically small molecules and gene editing, and expand on how such approaches can be considered in the context of the GENE TARGET framework.

RESULTS

Interrater agreement

Interrater agreement was measured across 5 raters who evaluated the same 20 gene-disorder pairs using the revised GENE TARGET framework (Table 1). For total GTS score, interrater agreement, measured by type (A, 1) intraclass correlation coefficient (ICC), was moderate between raters 1–3 (experienced) (0.67 [0.43, 0.84]), and poor between raters 4–5 (less experienced) (0.32 [−0.15, 0.66]) (Table S1). Figures 1A and 1B show interrater agreement across domains and criteria for raters 1–3 only. Raters 4–5 were excluded from further analysis given their poor agreement. The full results of interrater analysis across all 5 raters are shown in Table S1.

Raters 1–3 achieved substantial agreement, measured by Gwet's AC2, in all four domains: clinical (0.78 [0.67, 0.89]), pre-clinical (0.77 [0.70, 0.84]), genetic (0.65 [0.48, 0.82]), and ethical (AC2 = 0.64 [0.41, 0.88], Figure 1A). For criteria scores, 5 were in almost perfect agreement: early diagnosis (0.85 [0.68, 1.00]), natural history (0.95 [0.87, 1.00]), lack of available treatments (0.91 [0.80, 1.00]), genetic mechanism (0.86 [0.74, 0.98]), tools (0.82 [0.72, 0.91]); 4 were in substantial agreement: target population (0.80 [0.62, 0.98]), reversibility (0.63, [0.50, 0.76]), gene dosage (0.69 [0.49, 0.88]), and ethical considerations (0.63 [0.48, 0.79]); and one was in moderate agreement: endpoints (0.43 [0.19, 0.67]) (Figure 1B).

Final round of adjudication

The final round of adjudication (expert review by a clinical geneticist and scientist with deep expertise in preclinical modeling) of all 57 gene-disorder pairs led to 32 GTS score changes. At the level of criteria, 47 of 570 (8.2%) scores changed. The majority of score changes occurred in the pre-clinical domain (27/47; 57.4%), followed by the genetic (11/47; 23.4%), ethical (5/47; 10.6%), and clinical (4/47; 8.5%) domains.

GENE TARGET scores of 57 NDD gene-disorder pairs

The revised framework used to analyze 57 NDD gene-disorder pairs is shown in Table 1. Total GTS scores of these gene-disorder pairs are shown in Table 2, with full scorecards following Table S2. These tables reflect scores following the final round of adjudication. GTS scores ranged from 11 (*CSNK2B*, OMIM# 618732) to 37 (*STXBP1*, OMIM# 612164) with a mean GTS of 24.89 (Tables 2 and S2). The top ten scores were yielded by the following gene-disorder pairs: *STXBP1* (OMIM #612164); *ALDH5A1* (OMIM #271980); *UBE3A* (OMIM #105830); *SCN1A* (OMIM #607208); *SYNGAP1* (OMIM #612621); *EHMT1* (OMIM #610253); *SHANK3* (OMIM #606232); *CDKL5* (OMIM #300672); *PPP2R5D* (OMIM #616355); *VPS13B* (OMIM #216550), and *ZEB2* (OMIM #235730) were tied.

Cross-disorder analysis

A cross-disorder analysis of 57 gene-disorder pairs is represented by mean domain and adjusted criteria scores (ACSs, Figures 2A and 2B) and a heatmap (Figure 3). At the level of domains, ethical considerations yielded the highest mean score (8.33/10), followed by genetic mechanism (6.12/10), pre-clinical modeling (5.39/10), and clinical considerations (5.05/10, Figure 2A). Scores differed significantly across the four domains (Friedman test, $p < 0.001$). In post hoc

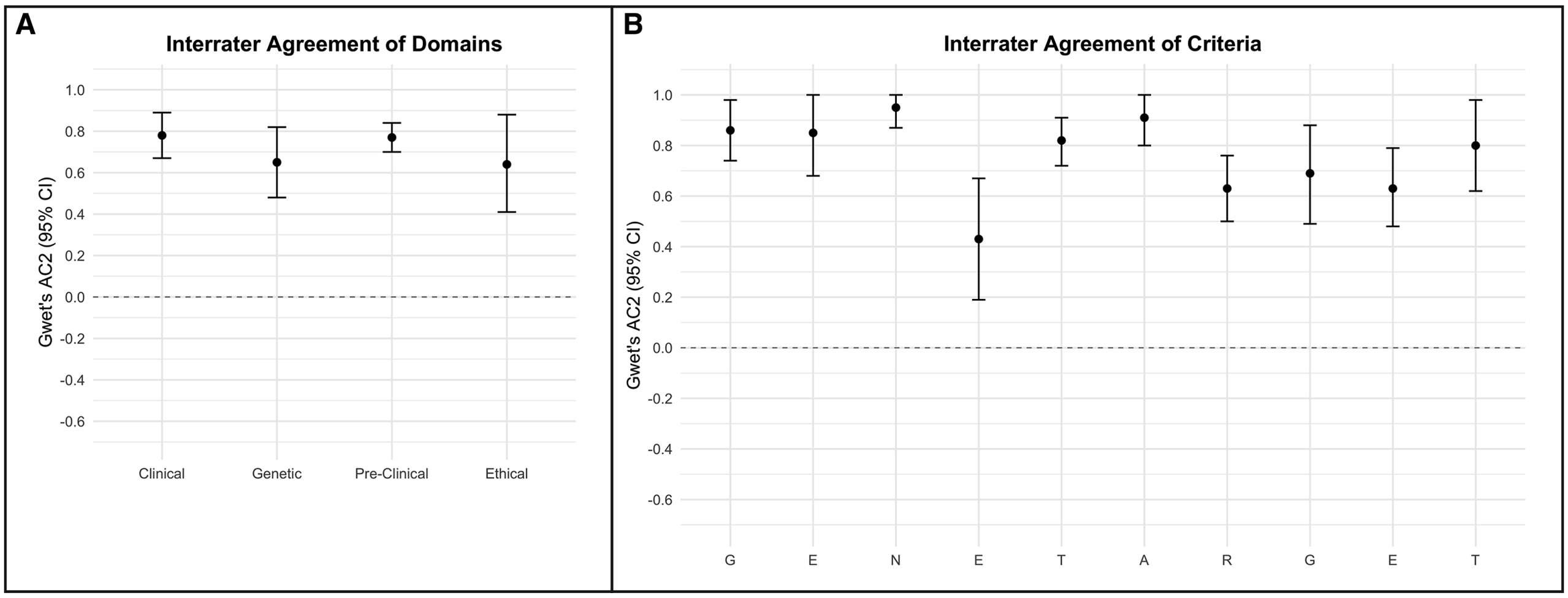

Figure 1. Interrater agreement (raters 1–3)

(A) Interrater agreement of the 4 GENE TARGET domains: clinical, genetic, pre-clinical, and ethical and (B) the ten GENE TARGET criteria: genetic mechanism, early diagnosis, natural history, endpoints, tools for target tissue at right time, availability of other treatments is limited, reversibility, gene dosage, ethics, and target population. A 95% confidence interval is reported for each Gwet's AC2 value.

Kappa 0.00–0.20, slight agreement; 0.21–0.40, fair agreement; 0.41–0.60, moderate agreement; 0.61–0.80, substantial agreement; 0.81–1.00, almost perfect agreement.

pairwise comparisons (Wilcoxon signed-rank tests, Holm-corrected), ethical considerations scored significantly higher than the genetic mechanism, clinical, and pre-clinical domains ($p < 0.001$ for all three comparisons), and genetic mechanism scored significantly higher than the clinical ($p = 0.001$) and pre-clinical ($p = 0.042$) domains, while clinical and pre-clinical scores did not differ significantly from each other ($p = 0.14$).

Mean ACS ranged from 0.29 to 0.89. The most common translational research gap, as defined by lowest mean ACS, was in the establishment of clinical trial endpoints, which yielded a mean ACS of 0.29 (Figure 2B). Gaps in natural history, early diagnosis, an understanding of dosage sensitivity, and a demonstration of reversibility were also common across disorders, all with a mean ACS less than or equal to 0.5. Lack of available treatments, and an engaged and accessible target population yielded the highest mean ACS, both scoring 0.89 (Figure 2B). Mean ACS differed significantly across the ten criteria (Friedman test, $p < 0.001$). In post hoc pairwise comparisons (Wilcoxon signed-rank tests, Holm-corrected), the establishment of clinical trial endpoints scored significantly lower than all nine other criteria ($p \leq 0.014$ for all comparisons), while lack of available treatments and an engaged and accessible target population each scored significantly higher than every other criterion ($p < 0.001$ for all comparisons) except ethical considerations and each other.

DISCUSSION

GENE TARGET was designed to facilitate evaluating the suitability of monogenic NDDs for genetic therapies, and to help identify gaps in the translational research pipeline both within and across conditions, with the ultimate purpose of improving quality of life outcomes for affected individuals. Implementing GENE TARGET in our Center's clinical-translational research evaluation process has enabled a more unbiased approach to disorder prioritization, such that limited resources for partnered research efforts may be strategically allocated.

Interrater agreement is at least moderate between trained raters; expert validation is recommended

GENE TARGET exhibits consistency between raters with adequate training. The three licensed genetic counselors with two or more years of experience in translational genomic medicine (raters 1–3) achieved higher levels of agreement in scores for each of the four domains, and nine of the ten criteria than the junior raters (raters 4–5), who had less than six months experience (Table S1). This highlights the importance of specialized training and experience in genetic medicine, rigorous interpretation of scientific literature, and appropriate use of recommended resources for optimal implementation of GENE TARGET. Others who utilize the framework, such as PAG leaders, may consider partnering with academic advisors or scientific advisory board members to help score criteria.

Given that the final round of adjudication resulted in an adjustment of 8.2% of criteria scores, validation of scores, particularly in the genetics and preclinical domains, by experts in clinical/molecular genetics, and preclinical modeling respectively, is recommended. Thus, equipped with targeted experience, genetic counselors are well-suited to perform initial assessments using this tool, supported by expert review for genetic mechanisms and preclinical domain-specific scoring.

Among the four domains, the ethical principles unsurprisingly exhibited the lowest level of interrater agreement. Weighing risks

Table 2. Total GTS scores of all 57 gene-disorder pairs reviewed

Gene-disorder pair	Total GTS score
ADNP (OMIM# 615873)	28
ALDH5A1 (OMIM# 271980)	36
ANKRD11 (OMIM# 148050)	19
ANKRD17 (OMIM# 619504)	22
ARID1B (OMIM# 135900)	27
ASH1L (OMIM# 617796)	28
ASXL3 (OMIM# 615485)	27
CASK (OMIM# 300749)	25
CDKL5 (OMIM# 300672)	31
CHAMP1 (OMIM# 616579)	22
CHD2 (OMIM# 615369)	29
CLCN4 (OMIM# 300114)	17
CSNK2A1 (OMIM# 617062)	19
CSNK2B (OMIM# 618732)	11
CTNNB1 (OMIM# 615075)	24
DDX3X (OMIM# 300958)	29
DEAF1 (OMIM# 615828)	17
DEAF1 (OMIM# 617171)	22
DLG4 (OMIM# 618793)	19
DNMT3A (OMIM# 615879)	18
DYNC1H1 (OMIM# 614563)	15
DYRK1A (OMIM# 614104)	25
EHMT1 (OMIM# 610253)	32
FOXP1 (OMIM# 613670)	28
GRIN2B (OMIM# 616139)	29
HEPACAM (OMIM# 613925)	19
HIVEP2 (OMIM# 616977)	25
HNRNPH2 (OMIM# 300986)	26
HNRNPK (OMIM# 616580)	20
HNRNPU (OMIM# 617391)	25
HUWE1 (OMIM# 309590)	22
IRF2BPL (OMIM# 618088)	24
KMT2A (OMIM# 605130)	23
KMT2C (OMIM# 617768)	20
MED13L (OMIM# 616789)	28
MEF2C (OMIM# 613443)	23
NEXMIF (OMIM# 300912)	27
PACS1 (OMIM# 615009)	27
PPP2R1A (OMIM# 616362)	18
PPP2R5D (OMIM# 616355)	31
RNU4-2 (OMIM# 620851)	18
SCN1A (OMIM# 607208)	34
SETD5 (OMIM# 615761)	23
SHANK3 (OMIM# 606232)	32
SLC6A1 (OMIM# 616421)	29
SMARCA4 (OMIM# 614609)	16
SON (OMIM# 617140)	28
STXBP1 (OMIM# 612164)	37
SYNGAP1 (OMIM# 612621)	33
TBR1 (OMIM# 606053)	24
TCF20 (OMIM # 618430)	21
TRIO (OMIM# 617061)	25
TRIO (OMIM# 618825)	24
UBE3A (OMIM# 105830)	35
VPS13B (OMIM# 216550)	30
ZEB2 (OMIM# 235730)	30
ZYMND11 (OMIM# 616083)	23

and benefits of a proposed treatment is, to an extent, inherently subjective, both in the context of this study and in real-world practice. This finding aligns closely with previous work characterizing the complex interplay of personal and professional factors—including experience, values, and beliefs—that influence clinical trial decision-making among gene therapy researchers.[11] At the level of the 10 criteria, evaluating the validity of clinical trial endpoints demonstrated the lowest level of interrater agreement. The disparities in defining what constitutes a potentially fit-for-purpose outcome for use in defining an endpoint, based on validity evidence from clinical outcome assessments, are somewhat expected. Identifying measures for monogenic NDDs that can show clinical significance for parents, caregivers, and clinicians, and ensuring these measures are valid, reliable, and responsive to change within the specific context of each condition is recognized as a major challenge.[12] In contrast, interrater agreement was highest for the criterion evaluating natural history data availability, followed closely by availability of treatments, and genetic mechanisms. This likely reflects the raters' professional backgrounds as genetic counselors engaged in natural history studies, the factual nature of these criteria, and the clarity of determining that disorders in our sample (46/57 in this study) have no available or investigational treatments.

Cross-disorder analysis shows clinical considerations as largest gap, followed by preclinical modeling and gene dosage

Across the 57 NDD gene-disorder pairs, clinical considerations yielded the lowest mean domain score and the three associated clinical criteria (validated endpoints, natural history, establishment of early diagnosis) yielded the lowest mean ACS (Figures 2A and 2B). This reflects the significant barriers to designing and executing successful longitudinal natural history studies including funding resources, infrastructure, and specialized expertise. In addition, participating in studies which typically require in-person neuropsychological assessments can place a significant burden on families.[13] Validating assessments takes time, and most assessments are not

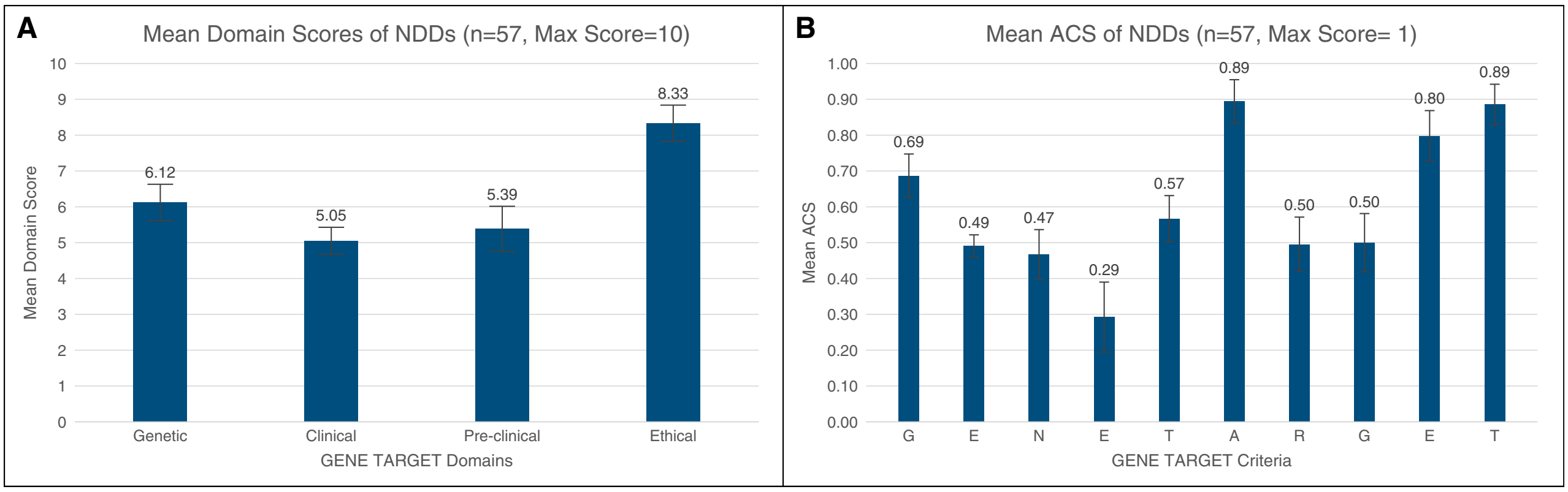

Figure 2. Mean domain and adjusted criteria scores of the 57 gene-disorder pairs

(A) Mean score of each domain: genetic, clinical, preclinical, and ethical considerations. (B) Mean ACS of each of the ten criteria: genetic mechanism, early diagnosis, natural history, endpoints, tools for target tissue, availability of other treatments is limited, reversibility, gene dosage, ethics, and target population. Error bars represent the 95% confidence interval of the mean across the 57 gene-disorder pairs.

universally appropriate for all NDDs and cognitive ages.[12,14,15] Natural history studies often identify outcome measures that lack suitability for the population, highlighting the need for additional studies and/or adaptation of existing measures.[16,17] Consequently, only eight of the gene-disorder pairs reviewed achieved the maximum score for endpoints (Table S2).

Within the clinical domain, establishment of an early diagnosis (ranked as the second-lowest scoring criterion) remains a persistent challenge. Rapid access to genomic sequencing remains a major, but addressable, barrier to treatment within the window of opportunity for phenotype reversal.[7,18] Since the publication of GENE TARGET, several large-scale studies of routine newborn genome sequencing have been initiated. In parallel, prenatal genome sequencing for fetal anomalies is increasingly being utilized.[19,20] The impact of prenatal/neonatal genome sequencing on potential for early treatment remains to be seen, but we anticipate substantial expansion in the range of disorders deemed amenable for treatment development as earlier therapeutic windows permitting phenotype reversal are identified.[21,22]

The preclinical domain ranked second lowest of the four domains, highlighting the challenge of understanding which cells in the brain need to be targeted for therapeutic rescue, how to target such cells, and at which point in neuronal development. Only five of the 57 gene-disorder pairs achieved the maximum score for the criterion “tools delivered to target tissue at the right time.” Since there are limitations to each of the model systems used for studying NDDs, this criterion will likely remain a challenge until we see convergence of evidence across multiple model systems. In CDKL5 deficiency disorder (CDD), for example, multiple groups have used rodent models to demonstrate that CDKL5 is not expressed in astrocytes but may be expressed in both glutamatergic and GABAergic neurons in different brain regions.[23–26] These results have been supported by induced pluripotent stem cells (iPSC)-derived models demonstrating neuron subtype-specific deficits in CDD neurons.[27–30] Only five disorders scored a maximum possible score for reversibility (reversibility and temporal window established), the criterion which could be considered the most essential for therapeutic development. Developing comprehensive models for monogenic disorders and using these to establish the temporal windows of reversibility remains challenging due to high costs, resource demands, and the intricate nature of replicating human brain functions across multiple systems.

An understanding of dosage windows was among the lowest-scoring criteria. As discussed in the original paper, this remains a barrier to therapeutic development, particularly for disorders with a haploinsufficiency mechanism for which a number of therapeutic approaches have been proposed including gene replacement therapy, wild-type upregulation using ASOs, and CRISPR activation of promoters to augment expression.[31–34] There is increasing computational evidence that many haploinsufficiency genes exhibit bidirectional dosage sensitivity where pathogenicity is likely to be observed with both over- and under-expression.[35] Cases of single gene duplications and resultant phenotypes (or lack thereof) are considered gold standard for the evaluation of triplosensitivity as per ClinGen Dosage Sensitivity Curations.[36] For most haploinsufficiency genes, however, such case-based examples do not exist.[35] Of the 57 gene-disorder pairs evaluated, 29 had a haploinsufficiency mechanism. Of these, only three were determined to have a definitively wide window of dosage tolerance, and 20 fell into the range of a suspected wide dosage range (no case-based evidence for triplosensitivity or tolerance for increased dosage in a haploinsufficiency gene). An accurate understanding of dosage remains a critical barrier for therapeutic approaches to haploinsufficiency disorders, as highlighted by analysis across gene-disorder pairs on our updated evaluation schema for this criterion.

Across the 57 gene-disorder pairs, the strongest criteria supporting therapeutic development were lack of availability of other treatments

Gene + Disorder	G	E	N	E	T	A	R	G	E	T
ADNP (OMIM# 615873)	0.33	0.50	0.33	0.67	1.00	0.50	0.75	0.50	1.00	1.00
ALDH5A1 (OMIM# 271980)	0.67	0.50	1.00	1.00	1.00	0.50	1.00	1.00	1.00	1.00
ANKRD11 (OMIM# 148050)	0.33	0.00	0.33	0.33	0.67	1.00	0.50	0.75	0.00	1.00
ANKRD17 (OMIM# 619504)	0.67	0.50	0.33	0.00	0.17	1.00	0.00	0.75	1.00	1.00
ARID1B (OMIM# 135900)	1.00	0.50	0.33	0.67	0.67	1.00	0.50	0.50	0.50	1.00
ASH1L (OMIM# 617796)	0.67	0.50	0.33	0.00	0.67	1.00	0.75	0.75	1.00	1.00
ASXL3 (OMIM# 615485)	0.67	0.50	0.33	0.00	0.67	1.00	0.50	0.75	1.00	1.00
CASK (OMIM# 300749)	1.00	0.50	0.33	0.00	0.33	1.00	0.75	0.75	0.50	1.00
CDKL5 (OMIM# 300672)	1.00	0.50	0.33	1.00	0.67	0.00	1.00	0.50	1.00	1.00
CHAMP1 (OMIM# 616579)	0.33	0.50	0.33	0.33	0.67	1.00	0.25	0.75	0.50	1.00
CHD2 (OMIM# 615369)	1.00	0.50	0.33	0.00	0.67	1.00	0.75	0.50	1.00	1.00
CLCN4 (OMIM# 300114)	0.50	0.50	0.33	0.00	0.33	1.00	0.25	0.00	0.50	1.00
CSNK2A1 (OMIM# 617062)	0.50	0.00	0.67	0.00	0.33	1.00	0.25	0.50	0.50	1.00
CSNK2B (OMIM# 618732)	0.33	0.50	0.33	0.00	0.00	1.00	0.00	0.00	0.50	0.50
CTNNB1 (OMIM# 615075)	0.67	0.50	0.33	0.33	0.67	1.00	0.50	0.50	0.50	1.00
DDX3X (OMIM# 300958)	1.00	0.50	0.33	0.00	0.67	1.00	0.50	0.75	1.00	1.00
DEAF1 (OMIM# 615828)	0.67	0.50	0.33	0.00	0.67	1.00	0.00	0.00	0.50	0.50
DEAF1 (OMIM# 617171)	0.67	0.50	0.33	0.00	0.67	1.00	0.50	0.00	1.00	0.50
DLG4 (OMIM# 618793)	0.67	0.50	0.33	0.00	0.17	1.00	0.25	0.50	0.50	1.00
DNMT3A (OMIM# 615879)	0.50	0.50	0.33	0.33	0.33	1.00	0.25	0.00	0.50	1.00
DYNC1H1 (OMIM# 614563)	0.00	0.50	0.33	0.00	0.33	1.00	0.50	0.00	0.50	1.00
DYRK1A (OMIM# 614104)	0.67	0.50	1.00	0.33	0.67	1.00	0.25	0.50	0.50	1.00
EHMT1 (OMIM# 610253)	1.00	0.50	0.33	1.00	0.67	1.00	0.75	0.50	1.00	1.00
FOXP1 (OMIM# 613670)	0.67	0.50	0.33	0.00	0.67	1.00	0.75	0.75	1.00	1.00
GRIN2B (OMIM# 613970)	0.67	0.50	0.33	1.00	0.67	0.50	0.75	0.50	1.00	1.00
HEPACAM (OMIM# 613925)	0.67	0.50	0.33	0.00	0.33	1.00	0.25	0.00	1.00	0.50
HIVEP2 (OMIM# 616977)	0.67	0.50	1.00	0.33	0.33	1.00	0.25	0.75	1.00	0.50
HNRNPH2 (OMIM# 300986)	1.00	0.50	1.00	0.33	0.67	1.00	0.50	0.00	0.50	1.00
HNRNPK (OMIM# 616580)	0.67	0.50	0.33	0.33	0.33	1.00	0.25	0.75	0.50	0.50
HNRNPU (OMIM# 617391)	0.67	0.50	0.33	0.00	0.33	1.00	0.50	0.75	1.00	1.00
HUWE1 (OMIM# 309590)	0.17	0.50	0.33	0.67	0.67	1.00	0.50	0.50	0.50	1.00
IRF2BPL (OMIM# 618088)	0.33	0.50	0.33	0.00	0.67	1.00	0.50	0.50	1.00	1.00
KMT2A (OMIM# 605130)	0.67	0.50	0.33	0.00	0.67	1.00	0.25	0.75	0.50	1.00
KMT2C (OMIM# 617768)	0.67	0.50	0.33	0.33	0.33	1.00	0.50	0.50	0.50	0.50
MED13L (OMIM# 616789)	0.67	0.50	0.33	0.33	0.67	1.00	0.75	0.50	1.00	1.00
MEF2C (OMIM# 613443)	0.67	0.50	0.33	0.00	0.67	1.00	0.50	0.50	0.50	1.00
NEXMIF (OMIM# 300912)	1.00	0.50	0.33	0.00	0.67	1.00	0.50	0.75	1.00	0.50
PACS1 (OMIM# 615009)	1.00	0.50	0.33	0.00	0.67	1.00	0.75	0.00	1.00	1.00
PPP2R1A (OMIM# 616362)	1.00	0.50	0.33	0.00	0.00	1.00	0.00	0.00	1.00	0.50
PPP2R5D (OMIM# 616355)	1.00	0.50	1.00	0.67	0.67	1.00	0.75	0.00	1.00	1.00
RNU4-2 (OMIM# 620851)	0.50	1.00	0.33	0.00	0.00	1.00	0.00	0.00	1.00	1.00
SCN1A (OMIM# 607208)	0.67	0.50	1.00	0.67	1.00	0.50	1.00	0.75	1.00	1.00
SETD5 (OMIM# 615761)	0.67	0.50	0.33	0.33	0.67	1.00	0.50	0.75	0.50	0.50
SHANK3 (OMIM# 606232)	0.67	0.50	1.00	1.00	0.67	0.50	1.00	0.50	1.00	1.00
SLC6A1 (OMIM# 616421)	0.67	0.50	0.33	0.67	0.67	0.50	0.75	0.75	1.00	1.00
SMARCA4 (OMIM# 614609)	0.67	0.50	0.33	0.00	0.33	1.00	0.25	0.00	0.50	0.50
SON (OMIM# 617140)	0.67	0.50	0.33	0.00	0.67	1.00	0.75	0.75	1.00	1.00
STXBP1 (OMIM# 612164)	1.00	0.50	1.00	1.00	1.00	0.50	1.00	0.75	1.00	1.00
SYNGAP1 (OMIM# 612621)	1.00	0.50	0.67	1.00	0.67	0.50	0.75	0.75	1.00	1.00
TBR1 (OMIM# 606053)	0.67	0.50	0.33	0.00	0.33	0.50	0.50	0.75	1.00	1.00
TCF20 (OMIM# 618430)	0.67	0.50	0.33	0.00	0.67	1.00	0.50	0.50	0.50	0.50
TRIO (OMIM# 618825)	0.67	0.50	0.33	0.00	0.67	1.00	0.25	0.50	1.00	1.00
TRIO (OMIM# 617061)	0.67	0.50	0.33	0.00	0.67	1.00	0.00	0.50	1.00	1.00
UBE3A (OMIM# 105830)	1.00	0.50	1.00	1.00	1.00	0.50	0.75	0.50	1.00	1.00
VPS13B (OMIM# 216550)	0.67	0.50	1.00	0.33	0.67	1.00	0.25	1.00	1.00	1.00
ZEB2 (OMIM# 235730)	0.67	0.50	0.67	0.67	0.67	1.00	0.75	0.50	1.00	1.00
ZYMND11 (OMIM# 616083)	0.67	0.50	0.33	0.00	0.33	1.00	0.25	1.00	1.00	0.50

Figure 3. Heatmap analysis of all 57 gene-disorder pairs reviewed with GENE TARGET

The adjusted criteria score (ACS) across all criteria, with darker colors correlating with higher scores, and lighter colors correlating with lower scores are shown for each gene. GENE TARGET criteria: genetic mechanism, early diagnosis, natural history, endpoints, tools for target tissue, availability of other treatments is limited, reversibility, gene dosage, ethics, and target population.

Table 3. Considerations for the application of GENE TARGET across multiple therapeutic modalities

Feature	Gene replacement therapy	ASOs/siRNA	Small molecules	Gene editing
Primary use case	loss-of-function, biallelic or mono-allelic	ASOs: loss-of function (upregulation/splicing)ASOs and siRNA: gain-of-function (knockdown)	modulating RNA or restoring/ inhibiting protein/pathway function	specific variant correction. read through of premature nonsense variants
Dosage control	difficult to titrate	titratable	highly titratable	requires high precision
Delivery	AAV vectors (IV or CNS administration)	IT or ICV injection, broad distribution	oral (ideal case)	LNPs or viral vectors, delivery efficiency is a bottleneck
Duration/reversibility	effectively permanent	reversible, chronic redosing	highly reversible, requires chronic redosing	permanent
Key risks	immunogenicity, overexpression toxicity, permanence	chronic invasive dosing, off-target hybridization, idiopathic toxicity	off-target biochemical effects, systemic toxicity	off-target editing, genomic instability, mosaicism
GENE TARGET implications	high scores needed for dosage tolerance and delivery tools, and careful consideration of risk benefit	dosage score is less penalized due to titration, high burden on patients, careful consideration of risk benefit	dosage score is less penalized, BBB penetrance critical for delivery score, risk benefit ratio may be more favorable given lower toxicities	high scrutiny of risk versus benefit and generalizability (ethical domain); delivery remains a challenge.

ASO, antisense oligonucleotides; siRNA, small interfering RNA; AAV, adeno-associated virus; IV, intravenous; CNS, central nervous system; ICV, intracerebroventricular; LNP, lipid nanoparticles; BBB, blood brain barrier.

and ethical considerations (weighing of risk and benefit). "Target populations are engaged and accessible" also scored high across disorders. However, there could have been selection bias, as those with active patient advocacy organizations, registries, and natural history studies were more likely to be selected through outreach or ongoing partnerships with the TNC.

While longitudinal tracking of specific gene-disorder pairs was not a specific goal of this work, *SHANK3*, which was scored in the original publication, was reevaluated in this project. Over the course of 4 years, the score for "natural history" shifted from a 2 to the maximum of 3, as did the score for "endpoints" (see Table S2 for references and rationale). Neither of these two criteria definitions was revised. Recently, a phase 1/2 trial for gene replacement therapy has been launched (NCT06662188), resulting in a drop in score of the criterion "availability of other safe and effective treatments is limited," from a 2 to a 1. In the updated version, this criterion was redefined to include treatments currently under evaluation. Thus, although the GTS increased by a single point (from 31 to 32), the more compelling narrative is found in the granular shifts of scores in individual criteria over time.

Considerations across therapeutic modalities

GENE TARGET was initially designed to assess the suitability of viral vector-mediated gene replacement therapy and RNA-targeted therapies, such as ASOs. However, the framework may also be applied—with some adjustments—to other approaches including RNA-targeting small molecules and gene editing, as well as mechanism-based small molecules that address the cause of disease at the protein or pathway level. Although some of the core criteria, such as the necessity for robust natural history data and validated endpoints, are universally applicable, the interpretation and relative importance of other criteria can vary significantly depending on the therapeutic modality. Both gene replacement therapy and ASO approaches, and their evaluation using GENE TARGET, have been previously discussed.[7] Here, we expand on the application of the framework to small molecules and gene editing technologies (Table 3).

Small molecules

Small molecules may be preferred due to lower cost, potential for less invasive administration, and broader biodistribution. This discussion is restricted to disease-modifying small molecules, which fall into two main categories. The first includes RNA-targeting small molecules, such as risdiplam for spinal muscular atrophy, which modulate gene expression at the RNA level and are considered genetic medicines.[37] The second includes molecules that restore the function of the affected protein or pathway, such as positive allosteric modulators for *KCNQ2* deficiency that aim to restore channel function.[38]

Similar to ASOs, the high degree of titratability inherent in small molecules mitigates concerns regarding the gene dosage criterion. The ability to easily adjust dosing regimens offers a significant advantage over gene replacement therapy, particularly for genes with a narrow or undefined therapeutic window.

The interpretation of the "tools delivered to target tissues" criterion differs substantially for small molecules. Unlike many nucleic acid-based medicines that require direct central nervous system (CNS) administration, small molecules have the potential to reach diverse tissue and cell types via systemic administration, such as oral dosing. Achieving a high score in this criterion is heavily dependent on the compound's ability to cross the blood brain barrier and achieve

therapeutic concentrations in relevant brain regions. Regarding ethical considerations, the potential for less invasive administration and the ability to rapidly withdraw treatment may favorably alter the risk-benefit calculation, support a higher score, and potentially expand the eligible target population.

Gene editing

Gene editing technologies, including CRISPR-Cas systems, base editing, and prime editing, offer the potential for permanent correction of pathogenic variants at the DNA level. The incorporation of these technologies into the GENE TARGET framework has been challenging due to the high allelic heterogeneity of most NDD genes, necessitating allele-specific tools. Typically, favorable consideration in GENE TARGET for gene editing approaches would be given only to monogenic disorders characterized by recurrent hotspot variants, and with a high degree of treatment generalizability for a patient population. However, prime-editing read through of premature termination codons to correct nonsense variants, which account for 24% of pathogenic variants in the ClinVar database, has been recently proposed as a therapeutic strategy.[39,40] This approach introduces a paradigm shift, suggesting the possibility of an allele-agnostic, and potentially disease-agnostic, gene editing approach.

Similar to challenges associated with therapeutic gene delivery, delivery also remains a significant hurdle for *in vivo* gene editing. The editing machinery must be delivered efficiently to the nucleus, typically by viral vectors or lipid nanoparticles. The challenge of achieving efficient editing in post mitotic neurons, which are the primary cell type implicated in many monogenic NDDs, is similar to that for gene replacement therapy. Adeno-associated virus (AAV) vectors, such as AAV9, are able to achieve significant expression in preclinical models for gene rescue in rodent brains, but the efficiency of delivery is significantly lower in primate brains, and novel capsids are being developed to address this gap.[41–48] In addition to the efforts to develop novel viral delivery tools, non-viral delivery methods, such as lipid nanoparticles, are also under development.[49–53] Optimization of these tools will improve translation of gene therapies for all NDDs.

Due to its permanence and the potential for unintended genomic alterations, gene editing currently carries the highest risk profile among genetic medicines. The ethical principles domain therefore requires significant scrutiny. At the present time, the risks of off-target editing, and on target but unintended edits may result in an unfavorable risk benefit ratio, even if the potential efficacy is high.

Study limitations

Despite the objectivity of the framework, each rater may harbor subconscious biases about particular gene-disorder pairs which could potentially impact ratings. Since the study was done at a single institution, there may also be some collective bias in the ratings, potentially limiting the generalizability of this data. A larger multi-institutional study with raters from a diverse range of professional backgrounds is a necessary future step to completely understand the interrater performance of the framework. Comparing these ratings with self-assessments from PAGs themselves would also be of interest.

A challenge for the GENE TARGET process is keeping pace with rapidly expanding diagnostic protocols, clinical translational literature, and pace of translational research. GENE TARGET scores are a snapshot and need to be updated on a regular basis based on evolving research and literature.

Selection of the 57 gene-disorder pairs included in this analysis was not random, so selection bias is present. It is likely that the range of scores for these 57 gene-disorder pairs is higher than for the total pool of NDD genes by the nature of how they were selected. In addition, these disorders represent a small fraction of the now over 2,000 unique genetic disorders of neurodevelopment. However, many of the concepts, such as challenges understanding gene dosage, in establishing endpoints, and collecting natural history data, are likely generalizable across disorders.

Future directions

The use and validation of GENE TARGET in settings beyond academic research, such as PAGs, biotechnology, and the pharmaceutical industry, remains an important area for development. PAGs and patients may be able to provide valuable insights into ethical considerations that resonate within the patient communities they represent.

Further training materials, such as videos, standard operating procedures, and a website with an online scoring calculator are future directions. Given the time-intensive and manual nature of these evaluations, use of artificial intelligence to mine literature and relevant databases for preliminary scoring is also a future direction and may address the question of scalability. This would allow for larger comparisons both within and across disorder types. Additionally, with such scalable technologies, a formal longitudinal project to evaluate the trajectory of scores over time for a group of genes is of interest in the future.

Conclusions

GENE TARGET, a novel framework encompassing clinical, preclinical, genetic, and ethical considerations for the systematic evaluation of genetic NDDs for therapeutic development, has been further refined, to more precisely discriminate between disorders with a more nuanced scoring rubric. While substantial agreement for most domain/criteria scores was demonstrated between raters who have had sufficient training, secondary review by experts with clinical/molecular genetics, and preclinical modeling expertise, is recommended. Clinical considerations (early diagnosis, understanding natural history, and establishment of endpoints) represent the most commonly identified gaps in therapeutic readiness across 57 NDD gene-disorder pairs, and that there is an urgent need for innovative, cost-effective, and scalable solutions to address this. Ethical considerations are the most difficult domain to evaluate objectively and tend to receive the most favorable scores overall, suggesting

Table 4. Rationale and examples for revisions to GENE TARGET scoring

Revised criterion	Rationale for revision(s)	Example
Genetic mechanism is understood and amenable	a gene may display one or more special characteristics (recurrent hotspot mutations, non-productive transcripts, genomic imprinting, X-linked dominant inheritance, and paralog genes), even when an understanding of the underlying genetic mechanism of disease is emerging; additionally, it is recognized that there are grades of evidence for underlying mechanisms	the underlying mechanism has not yet been definitively established for ReNU syndrome [OMIM: 620851], although toxic gain of function is strongly suspected; there is a recurrent variant, (n.64_65insT) which accounts for 70% of the variant spectrum, which is considered a special mechanism[54]; score assigned = 3
Early diagnosis is typical	rather than scoring solely on the typical age of diagnosis, the age of clinical presentation is now included; this accounts for changing thresholds for genetic testing, and for the concept that the *potential* for early diagnosis should be recognized	ReNU syndrome [OMIM: 620851] typically presents with neonatal hypotonia, thus there is the potential for early diagnosis[54]; score assigned = 2
Tools to deliver to target tissues at the right time	substantial changes were made to more precisely define target tissue to include the level of brain region and cell type, and stage of neuronal development, and to in parallel recognize the development and availability of preclinical models	iPSCs and mouse models are currently under development for *ANKRD17* [OMIM: 619504] but are not yet publicly available; cell type and brain region remain undefined; score assigned = 1
Availability of other safe and effective treatments is limited	the increasingly common scenario of disease altering treatments being evaluated in clinical trials is now included, rather than just the availability of an approved treatment	for *STXBP1* [OMIM: 612164], FDA announced fast track designation in May 2025 for an IV-administered gene therapy (PAG website); Phenylbutyrate is being investigated as a treatment for monogenic DEEs (clinicaltrials.gov: NCT04937062); score assigned = 1
Reversibility has been demonstrated	the scores now incorporate the availability of publicly available preclinical resources to conduct reversibility experiments. In addition, the establishment of a preclinical endpoint is also included; these yield new mid-range scores which are key intermediate steps in the establishment of reversibility in a preclinical model system	two variants have been functionally rescued, but without demonstration of phenotypic rescue[55]; however, Clcn4 knockout and Clcn4 A549V point mutation rat models are publicly available to purchase through INFRAFRONTIER[56]; score assigned = 1
Gene is tolerant to dosage changes	for disorders with a haploinsufficiency mechanism, documented cases of single gene duplications and resultant phenotypes (or lack thereof) are considered gold standard for the evaluation of dosage tolerance/intolerance as per the ClinGen dosage sensitivity curation (Rehm et al.[36]). Most of these genes, such case base examples do not exist; the new scoring category of "wide dosage windows suspected" was added to capture this	haploinsufficiency is the mechanism for *HNRNPU* [OMIM: 617391], curated by ClinGen Dosage Sensitivity group, but there is no current evidence of triplosensitivity[36]; the duplications in association with NDD phenotypes reported in Decipher this gene are large, multi-gene CNVs; score assigned = 3
Ethical principles have been considered	considerations now include situations where ethical principles are deemed unfavorable	none of the gene-disorder pairs reviewed scored a 0, thus no relevant examples

that ethical arguments alone are unlikely to effectively justify prioritizing one disorder over another. Additionally, specific adjustments needed for the application of the GENE TARGET framework to small molecules and gene editing modalities have been reviewed.

MATERIALS AND METHODS

Revision of scoring schema and resource guide

Through systematic implementation of GENE TARGET, limitations in the scoring categories were identified. The authors, with multidisciplinary expertise in developmental medicine, child neurology, and clinical genetics, iteratively revised the scoring schema following round table discussions to reach consensus. The updated scoring framework, as shown in Table 1, includes seven new mid-range scores, and amendments to the 10 existing score definitions. The rationale for each criterion scoring change is shown in Table 4. To increase the rigor, consistency, and objectivity of the framework, a resource guide was developed. Serving as a standard operating procedure manual for raters, this guide specifies the reliable and trusted databases, sources, and websites to reference in evaluation and scoring (Table 5). The guide includes publicly available, reputable resources such as GeneReviews for clinical considerations (for example, typical age of presentation), ClinGen Curations (for example, for genetic mechanism and dosage sensitivity), clinicaltrials.gov (for natural history, endpoints, and treatments under evaluation) in addition to primary peer-reviewed literature.[36,57] PAG websites are also included, given their critical role in evaluating

Table 5. GENE TARGET resource guide to aid in evaluation of gene-disorder pairs

	Criteria	Resource guide[a]
G	genetic mechanism is understood	GeneReviews[b], OMIM[c], primary literature, ClinGen Dosage Sensitivity[d], Decipher[e]
E	early diagnosis is typical	GeneReviews, OMIM, primary literature
N	natural history is understood	primary literature,[f] clinicaltrials.gov
E	endpoints are validated and meaningful	primary literature, clinicaltrials.gov;
T	tools deliver to target tissue at the right time	primary literature, GTEx[g] and gnomAD[h] (for expression information), PAG websites, Jackson Laboratory[i], Coriell Institute[j]
A	availability of other safe and effective treatments is limited	Clinicaltrials.gov, primary literature
R	reversibility has been demonstrated	primary literature, clinicaltrials.gov
G	gene is tolerant to dosage changes	ClinGen dosage sensitivity, Decipher, primary literature, ClinVar[i]
E	ethical principles have been considered	ClinVar, ClinGen dosage sensitivity, GeneReviews, primary literature, OMIM
T	target populations are accessible and engaged	PAG websites, Facebook, GeneReviews, NORD[k]

[a]PAG websites can be a helpful resource to help identify additional information.
[b]GeneReviews: https://www.ncbi.nlm.nih.gov/books/NBK1116/.
[c]OMIM: https://omim.org/.
[d]ClinGen: https://www.clinicalgenome.org/.
[e]DECIPHER: https://www.deciphergenomics.org/.
[f]ClinicalTrials.gov: https://clinicaltrials.gov/.
[g]GTEx Portal: https://www.gtexportal.org/home/.
[h]gnomAD: https://gnomad.broadinstitute.org/.
[i]Jackson Laboratory: https://www.jax.org/jax-mice-and-services/find-mice.
[j]Coriell Institute: https://www.coriell.org/.
[k]NORD: https://rarediseases.org/.

the "target populations are engaged and accessible" criterion, and their frequent inclusion of preclinical resources available to researchers. No "data packets" were provided to raters.

Gene-disorder pair selection

All gene-disorder pairs included in this analysis are definitively implicated in monogenic disorders in which neurodevelopmental manifestations are a key characteristic. While some disorders have extraneurological manifestations, the presumed target organ was the central nervous system for all conditions.

Recognizing that some genes are implicated in more than one disorder, evaluations are for gene-disorder entities (as defined by a single OMIM #, a unifying molecular mechanism, and pattern of inheritance), rather than genes, as discussed in our initial publication. Gene-disorder pairs that TNC faculty and affiliate faculty were actively researching and thus for which we had subject expertise were prioritized for selection. Additional genetic disorders were nominated by both research and advocacy collaborators. The resulting 57 gene-disorder pairs are listed in Table 2.

Interrater agreement

Five raters each evaluated 20 of the 57 NDD gene-disorder pairs using the revised GENE TARGET framework and resource guide. Raters 1, 2, and 3 were licensed genetic counselors with similar experience (at least 2 years) in translational genomic medicine. Raters 4 and 5 were a genetic counseling assistant and a genetic counseling student, respectively, with limited experience in this field (less than six months). A time limit of 90 min per review was set. Evaluations occurred within a 6-months time frame (March-August 2024) to limit the possibility of newly published information impacting scoring of a given gene-disorder pair.

Interrater agreement was calculated for raters 1–3 and raters 4–5, respectively, for total GTS score, and for the four domain and ten criteria scores. The type (A, 1) ICC was estimated to measure the interrater agreement between the raters for continuous variables (total score).[58] The type (A, 1) ICC was computed for all raters and all pairs of two raters. The interrater agreement of ordinal scores between any pair (two) of the raters was evaluated by Gwet's AC2 using ordinal weights.[59,60] A 95% confidence interval (CI) was reported for each Gwet's AC2 value. Agreement across raters 1–5 was not evaluated due to differences in experience level in the field. Pairwise interrater agreement (with CI) was also calculated for raters 1–3.

The estimated value of the type (A, 1) ICC is interpreted as follows: an ICC value less than 0.50 is indicative of poor reliability; between 0.50 and 0.75, moderate reliability; between 0.75 and 0.90, good reliability; greater than 0.90, excellent reliability.[61] Landis and Koch guidelines were followed for interpretation of Gwet's AC2: a kappa

between 0.00 and 0.20 indicated slight agreement; between 0.21 and 0.40, fair agreement; between 0.41 and 0.60, moderate agreement; between 0.61 and 0.80, substantial agreement; greater than 0.80, almost perfect agreement.[62]

To resolve any scoring discrepancies of 20 gene-disorder pairs scored in the interrater analysis, raters 1–3 had round-table discussions to reach consensus scores.

Scoring 57 NDD gene-disorder pairs

57 total gene-disorder pairs were reviewed. As noted above, 20 of the 57 were evaluated by 5 raters each with subsequent consensus review. The remaining 37 gene-disorder pairs were evaluated by a single rater (raters 1–3 only). All evaluations were completed between November 2023 and July 2025.

Finally, for all 57 gene-disorder pairs, all scores were reviewed for a final round of adjudication by a clinical geneticist, together with input from a scientist with expertise in preclinical modeling to ensure validation prior to cross disorder analysis.

Cross-disorder analysis

Common research gaps and strengths along the translational pipeline across the 57 gene-disorder pairs were characterized through analysis of domain and criterion scores. First, for each criterion, the score was divided by the maximum possible score for that particular criterion. These ACS ranged from 0 to 1, with 1 representing the highest score for a given criterion. For example, a score of 2 for "genetic mechanism is understood and amenable" would be divided by the maximum possible score of 6, generating an ACS of 0.33. A heatmap was generated demonstrating ACS across gene-disorder pairs. Next, we calculated the mean ACS for each of the 10 criteria across the 57 gene-disorder pairs reviewed. Values again ranged from 0 to 1, with 1 representing the most translational progress for a given criterion. Mean ACS were plotted on a bar graph against criteria. Mean domain scores (equally weighted with a maximum of 10 points each) across all 57 gene-disorder pairs were also calculated. Differences across the 4 mean domain scores and across the 10 mean ACS, respectively, were assessed using Friedman tests. Where the Friedman test was significant, post hoc pairwise comparisons were performed using Wilcoxon signed-rank tests with Holm correction for multiple comparisons. Statistical significance was defined as a corrected $p < 0.05$.

DATA AND CODE AVAILABILITY

Data supporting this study are included within the manuscript and its supplemental information.

ACKNOWLEDGMENTS

We thank the Rosamund Stone Zander and Hansjörg Wyss TNC for its resources and intellectual contributions as well as the IDDRC Clinical Translational Core (NIH P50 HD105351). We also acknowledge the Developmental Synaptopathies Consortium (U54NS092090), part of the Rare Diseases Clinical Research Network (RDCRN), an initiative of the Office of Rare Diseases Research (ORDR) within the National Center for Advancing Translational Sciences (NCATS). This consortium is funded through a collaborative effort between NCATS and the National Institute of Neurological Disorders and Stroke (NINDS) of the National Institutes of Health.

AUTHOR CONTRIBUTIONS

The project was conceptualized by R.B.D., A.S., Z.F., K.A.D., S.J.B., P.T., L.P., J.H., E.D.B., M.S., and M.C. Data was curated by R.B.D. Formal analysis was completed by R.B.D., B.Z., and M.E.R. Initial investigation and scoring of gene-disorder pairs was completed by R.B.D., A.S., Z.F., H.O., and C.B. Methodology was defined by R.B.D., A.S., M.C., B.Z., and M.E.R. M.C. provided supervision of the project. The original draft was written by R.B.D., M.C., J.H., and E.D.B. All authors reviewed and edited subsequent drafts.

DECLARATION OF INTERESTS

The authors declare no competing interests.

DECLARATION OF GENERATIVE AI AND AI-ASSISTED TECHNOLOGIES IN THE MANUSCRIPT PREPARATION PROCESS

During the preparation of this manuscript, the authors used Google Gemini for language editing, including rephrasing and polishing of portions of the text. All AI-assisted output was reviewed and edited by the authors, who take full responsibility for the final content.

SUPPLEMENTAL INFORMATION

Supplemental information can be found online at https://doi.org/10.1016/j.omta.2026.201871.

REFERENCES

1. Lerche, H., Hedrich, U.B., and Wuttke, T.V. (2025). Gene-replacement therapy in neurodevelopmental disorders: progress and challenges. J. Clin. Investig. *135*, e188703. https://doi.org/10.1172/jci188703.

2. Quilón, P.G., Volpedo, G., Cappato, S., Ferrera, L., Zara, F., Bocciardi, R., Riva, A., and Striano, P. (2024). Antisense oligonucleotides as a precision therapy for developmental and epileptic encephalopathies. CNS Neurosci. Ther. *30*, e70050. https://doi.org/10.1111/cns.70050.

3. Müller, L., Jacobsen, B., and Nürnberg, A. (2025). Risdiplam: A Small Molecule mRNA Splice Modifier Approved to Treat SMA Disease. Toxicol. Pathol. *53*, 695–699. https://doi.org/10.1177/01926233251392863.

4. Navarrete-Opazo, A.A., Singh, M., Tisdale, A., Cutillo, C.M., and Garrison, S.R. (2021). Can you hear us now? The impact of health-care utilization by rare disease patients in the United States. Genet. Med. *23*, 2194–2201. https://doi.org/10.1038/s41436-021-01241-7.

5. Brooks, P.J., Grady, A.C., Groft, S., Ho, L., Lumsden, J., Shah, M., Sid, E., Xu, Y., Tisdale, A., Dickens, J., et al. (2024). The division of rare diseases research innovation at the national center for advancing translational sciences, NIH: mission, history, and current research activities. Rare Dis. Orphan Drug. J. *3*, 15. https://doi.org/10.20517/rdodj.2023.27.

6. Shellhaas, R.A., deVeber, G., and Bonkowsky, J.L.; Child Neurology Society Research Committee (2021). Gene-Targeted Therapies in Pediatric Neurology: Challenges and Opportunities in Diagnosis and Delivery. Pediatr. Neurol. *125*, 53–57. https://doi.org/10.1016/j.pediatrneurol.2021.09.011.

7. Chopra, M., Modi, M.E., Dies, K.A., Chamberlin, N.L., Buttermore, E.D., Brewster, S.J., Prock, L., and Sahin, M. (2022). GENE TARGET: A framework for evaluating Mendelian neurodevelopmental disorders for gene therapy. Mol. Ther. Methods Clin. Dev. *27*, 32–46. https://doi.org/10.1016/j.omtm.2022.08.007.

8. Lee, H.H.C., Latzer, I.T., Bertoldi, M., Gao, G., Pearl, P.L., Sahin, M., and Rotenberg, A. (2024). Gene replacement therapies for inherited disorders of neurotransmission: Current progress in succinic semialdehyde dehydrogenase deficiency. J. Inherit. Metab. Dis. *47*, 476–493. https://doi.org/10.1002/jimd.12735.

9. SCN8A Research Consortium (2025). A research roadmap for SCN8A-related disorders: addressing knowledge gaps and aligning research priorities across stakeholders. Orphanet J. Rare Dis. *20*, 444. https://doi.org/10.1186/s13023-025-03672-w.

10. Major, R.M., and Juengst, E.T. (2025). Prenatal gene editing for neurodevelopmental diseases: Ethical considerations. Am. J. Hum. Genet. *112*, 201–214. https://doi.org/10.1016/j.ajhg.2025.01.003.

11. Deakin, C.T., Alexander, I.E., Hooker, C.A., and Kerridge, I.H. (2013). Gene therapy researchers' assessments of risks and perceptions of risk acceptability in clinical trials. Mol. Ther. *21*, 806–815. https://doi.org/10.1038/mt.2012.230.

12. Farmer, C., Kaat, A.J., Berry-Kravis, E., and Thurm, A. (2022). Chapter One - Psychometric perspectives on developmental outcome and endpoint selection in treatment trials for genetic conditions associated with neurodevelopmental disorder. In International Review of Research in Developmental Disabilities, A.J. Esbensen and E.K. Schworer, eds. (Academic Press), pp. 1–39. https://doi.org/10.1016/bs.irrdd.2022.05.001.

13. U.S. Food and Drug Administration (FDA) (2019). Rare Diseases: Natural History Studies for Drug Development Guidance for Industry (U.S. Food and Drug Administration (FDA)).

14. Thurm, A., Kelleher, B., and Wheeler, A. (2020). Outcome Measures for Core Symptoms of Intellectual Disability: State of the Field. Am. J. Intellect. Dev. Disabil. *125*, 418–433. https://doi.org/10.1352/1944-7558-125.6.418.

15. U.S. Food and Drug Administration (FDA) (2025). Patient-FOcused Drug Development: Selecting, Developing, or Modifying Fit-For-Purpose Clinical Outcome Assessments (U.S. Food and Drug Administration (FDA)).

16. Valluripalli Soorya, L., Brune, C.W., Farmer, C.A., Ocampo, E.V., Berger, N.I., Pearson, D.A., Busch, R.M., Klaas, P., Siper, P., Currans, K., et al. (2025). Characterizing Developmental and Behavioral Profiles in Developmental Synaptopathies to Inform Clinical Trial Endpoints. Am. J. Intellect. Dev. Disabil. *130*, 414–437. https://doi.org/10.1352/1944-7558-130.5.414.

17. Berg, A.T., Ludwig, N.N., Wojnaroski, M., Chapman, C.A.T., Hommer, R., Conecker, G., Hecker, J.Z., and Downs, J. (2024). FDA Patient-Focused Drug Development Guidances: Considerations for Trial Readiness in Rare Developmental and Epileptic Encephalopathies. Neurology *102*, e207958. https://doi.org/10.1212/wnl.0000000000207958.

18. Davidson, B.L., Gao, G., Berry-Kravis, E., Bradbury, A.M., Bönnemann, C., Buxbaum, J.D., Corcoran, G.R., Gray, S.J., Gray-Edwards, H., Kleiman, R.J., et al. (2022). Gene-based therapeutics for rare genetic neurodevelopmental psychiatric disorders. Mol. Ther. *30*, 2416–2428. https://doi.org/10.1016/j.ymthe.2022.05.014.

19. Jeanne, M., and Chung, W.K. (2025). Prenatal genomic sequencing: Navigating uncertainty. Semin. Perinatol. *49*, 152058. https://doi.org/10.1016/j.semperi.2025.152058.

20. van den Veyver, I.B., and Eng, C.M. (2015). Genome-Wide Sequencing for Prenatal Detection of Fetal Single-Gene Disorders. Cold Spring Harb. Perspect. Med. *5*, a023077. https://doi.org/10.1101/cshperspect.a023077.

21. Carli, D., Quarello, P., Porta, F., Cagnazzo, C., Zucchetti, G., Proto, C.F., Gianasso, R., Biamino, E., Carbonara, C., Coscia, A., et al. (2025). A Genomic Sequencing Approach to Newborn Mass Screening and Its Opportunities. JAMA Netw. Open *8*, e2538198. https://doi.org/10.1001/jamanetworkopen.2025.38198.

22. Ziegler, A., Koval-Burt, C., Kay, D.M., Suchy, S.F., Begtrup, A., Langley, K.G., Hernan, R., Amendola, L.M., Boyd, B.M., Bradley, J., et al. (2025). Expanded Newborn Screening Using Genome Sequencing for Early Actionable Conditions. JAMA *333*, 232–240. https://doi.org/10.1001/jama.2024.19662.

23. Silvestre, M., Dempster, K., Mihaylov, S.R., Claxton, S., and Ultanir, S.K. (2024). Cell type-specific expression, regulation and compensation of CDKL5 activity in mouse brain. Mol. Psychiatry *29*, 1844–1856. https://doi.org/10.1038/s41380-024-02434-7.

24. Zhu, Z.A., Li, Y.Y., Xu, J., Xue, H., Feng, X., Zhu, Y.C., and Xiong, Z.Q. (2023). CDKL5 deficiency in adult glutamatergic neurons alters synaptic activity and causes spontaneous seizures via TrkB signaling. Cell Rep. *42*, 113202. https://doi.org/10.1016/j.celrep.2023.113202.

25. Tang, S., Wang, I.T.J., Yue, C., Takano, H., Terzic, B., Pance, K., Lee, J.Y., Cui, Y., Coulter, D.A., and Zhou, Z. (2017). Loss of CDKL5 in Glutamatergic Neurons Disrupts Hippocampal Microcircuitry and Leads to Memory Impairment in Mice. J. Neurosci. *37*, 7420–7437. https://doi.org/10.1523/jneurosci.0539-17.2017.

26. Pizzo, R., Gurgone, A., Castroflorio, E., Amendola, E., Gross, C., Sassoè-Pognetto, M., and Giustetto, M. (2016). Lack of Cdkl5 Disrupts the Organization of Excitatory and Inhibitory Synapses and Parvalbumin Interneurons in the Primary Visual Cortex. Front. Cell. Neurosci. *10*, 261. https://doi.org/10.3389/fncel.2016.00261.

27. Glass, M.R., Whye, D., Anderson, N.C., Wood, D., Makhortova, N.R., Polanco, T., Kim, K.H., Donovan, K.E., Srinivasan, G.R., Vaccaro, L., et al. (2025). Excitatory cortical neurons from CDKL5 deficiency disorder patient-derived organoids show early hyperexcitability not identified in neurogenin2 induced neurons. Neurobiol. Dis. *215*, 107093. https://doi.org/10.1016/j.nbd.2025.107093.

28. La Montanara, P., Hervera, A., Baltussen, L.L., Hutson, T.H., Palmisano, I., De Virgiliis, F., Kong, G., Chadwick, J., Gao, Y., Bartus, K., et al. (2020). Cyclin-dependent-like kinase 5 is required for pain signaling in human sensory neurons and mouse models. Sci. Transl. Med. *12*, eaax4846. https://doi.org/10.1126/scitranslmed.aax4846.

29. Wu, W., Yao, H., Negraes, P.D., Wang, J., Trujillo, C.A., de Souza, J.S., Muotri, A.R., and Haddad, G.G. (2022). Neuronal hyperexcitability and ion channel dysfunction in CDKL5-deficiency patient iPSC-derived cortical organoids. Neurobiol. Dis. *174*, 105882. https://doi.org/10.1016/j.nbd.2022.105882.

30. Negraes, P.D., Trujillo, C.A., Yu, N.K., Wu, W., Yao, H., Liang, N., Lautz, J.D., Kwok, E., McClatchy, D., Diedrich, J., et al. (2021). Altered network and rescue of human neurons derived from individuals with early-onset genetic epilepsy. Mol. Psychiatry *26*, 7047–7068. https://doi.org/10.1038/s41380-021-01104-2.

31. Lim, K.H., Han, Z., Jeon, H.Y., Kach, J., Jing, E., Weyn-Vanhentenryck, S., Downs, M., Corrionero, A., Oh, R., Scharner, J., et al. (2020). Antisense oligonucleotide modulation of non-productive alternative splicing upregulates gene expression. Nat. Commun. *11*, 3501. https://doi.org/10.1038/s41467-020-17093-9.

32. Han, Z., Chen, C., Christiansen, A., Ji, S., Lin, Q., Anumonwo, C., Liu, C., Leiser, S.C., Aznarez, I., Aznarez, I., et al. (2020). Antisense oligonucleotides increase Scn1a expression and reduce seizures and SUDEP incidence in a mouse model of Dravet syndrome. Sci. Transl. Med. *12*, eaaz6100. https://doi.org/10.1126/scitranslmed.aaz6100.

33. Ghahremani, S., Kanwal, A., Pettinato, A., Ladha, F., Legere, N., Thakar, K., Zhu, Y., Tjong, H., Wilderman, A., Stump, W.T., et al. (2024). CRISPR Activation Reverses Haploinsufficiency and Functional Deficits Caused by TTN Truncation Variants. Circulation *149*, 1285–1297. https://doi.org/10.1161/circulationaha.123.063972.

34. Matharu, N., Rattanasopha, S., Tamura, S., Maliskova, L., Wang, Y., Bernard, A., Hardin, A., Eckalbar, W.L., Vaisse, C., and Ahituv, N. (2019). CRISPR-mediated activation of a promoter or enhancer rescues obesity caused by haploinsufficiency. Science *363*, eaau0629. https://doi.org/10.1126/science.aau0629.

35. Collins, R.L., Glessner, J.T., Porcu, E., Lepamets, M., Brandon, R., Lauricella, C., Han, L., Morley, T., Niestroj, L.M., Ulirsch, J., et al. (2022). A cross-disorder dosage sensitivity map of the human genome. Cell *185*, 3041–3055.e25. https://doi.org/10.1016/j.cell.2022.06.036.

36. Rehm, H.L., Berg, J.S., Brooks, L.D., Bustamante, C.D., Evans, J.P., Landrum, M.J., Ledbetter, D.H., Maglott, D.R., Martin, C.L., Nussbaum, R.L., et al. (2015). ClinGen–the Clinical Genome Resource. N. Engl. J. Med. *372*, 2235–2242. https://doi.org/10.1056/NEJMsr1406261.

37. Baranello, G., Darras, B.T., Day, J.W., Deconinck, N., Klein, A., Masson, R., Mercuri, E., Rose, K., El-Khairi, M., Gerber, M., et al. (2021). Risdiplam in Type 1 Spinal Muscular Atrophy. N. Engl. J. Med. *384*, 915–923. https://doi.org/10.1056/NEJMoa2009965.

38. Gunthorpe, M.J., Large, C.H., and Sankar, R. (2012). The mechanism of action of retigabine (ezogabine), a first-in-class K+ channel opener for the treatment of epilepsy. Epilepsia *53*, 412–424. https://doi.org/10.1111/j.1528-1167.2011.03365.x.

39. Landrum, M.J., Lee, J.M., Riley, G.R., Jang, W., Rubinstein, W.S., Church, D.M., and Maglott, D.R. (2014). ClinVar: public archive of relationships among sequence variation and human phenotype. Nucleic Acids Res. *42*, D980–D985. https://doi.org/10.1093/nar/gkt1113.

40. Pierce, S.E., Erwood, S., Oye, K., An, M., Krasnow, N., Zhang, E., Raguram, A., Seelig, D., Osborn, M.J., and Liu, D.R. (2025). Prime editing-installed suppressor tRNAs for disease-agnostic genome editing. Nature *648*, 191–202. https://doi.org/10.1038/s41586-025-09732-2.

41. Lee, N.K., Na, D.L., Kim, J.W., Lee, B., Kim, H.J., Jang, H., Lee, J., and Chang, J.W. (2025). Evaluation of AAV transduction efficiency via multiple delivery

routes: Insights from peripheral and central nervous system analysis. Neuroscience *573*, 96–103. https://doi.org/10.1016/j.neuroscience.2025.03.026.

42. Chandran, J., Chowdhury, E.A., Perkinton, M., Jamier, T., Sutton, D., Wu, S., Dobson, C., Shah, D.K., Chessell, I., and Meno-Tetang, G.M.L. (2023). Assessment of AAV9 distribution and transduction in rats after administration through Intrastriatal, Intracisterna magna and Lumbar Intrathecal routes. Gene Ther. *30*, 132–141. https://doi.org/10.1038/s41434-022-00346-1.

43. Hammond, S.L., Leek, A.N., Richman, E.H., and Tjalkens, R.B. (2017). Cellular selectivity of AAV serotypes for gene delivery in neurons and astrocytes by neonatal intracerebroventricular injection. PLoS One *12*, e0188830. https://doi.org/10.1371/journal.pone.0188830.

44. Bey, K., Ciron, C., Dubreil, L., Deniaud, J., Ledevin, M., Cristini, J., Blouin, V., Aubourg, P., and Colle, M.A. (2017). Efficient CNS targeting in adult mice by intrathecal infusion of single-stranded AAV9-GFP for gene therapy of neurological disorders. Gene Ther. *24*, 325–332. https://doi.org/10.1038/gt.2017.18.

45. Okai, T., Sato, S., Yasuno, H., Nakayama, M., Yamamoto, S., Sjöqvist, S., Otake, K., Nakashima, M., Deshpande, M., Galbreath, E., et al. (2025). Biodistribution of AAV1, AAV5, AAV9, and AAVDJ serotypes after intra-cisterna magna delivery in non-human primates. Mol. Ther. Methods Clin. Dev. *33*, 101593. https://doi.org/10.1016/j.omtm.2025.101593.

46. Chen, X., Wolfe, D.A., Bindu, D.S., Zhang, M., Taskin, N., Goertsen, D., Shay, T.F., Sullivan, E.E., Huang, S.F., Ravindra Kumar, S., et al. (2023). Functional gene delivery to and across brain vasculature of systemic AAVs with endothelial-specific tropism in rodents and broad tropism in primates. Nat. Commun. *14*, 3345. https://doi.org/10.1038/s41467-023-38582-7.

47. Stanton, A.C., Lagerborg, K.A., Tellez, L., Krunnfusz, A., King, E.M., Ye, S., Solomon, I.H., Tabebordbar, M., and Sabeti, P.C. (2023). Systemic administration of novel engineered AAV capsids facilitates enhanced transgene expression in the macaque CNS. Med *4*, 31–50.e8. https://doi.org/10.1016/j.medj.2022.11.002.

48. Chen, X., Ravindra Kumar, S., Adams, C.D., Yang, D., Wang, T., Wolfe, D.A., Arokiaraj, C.M., Ngo, V., Campos, L.J., Griffiths, J.A., et al. (2022). Engineered AAVs for non-invasive gene delivery to rodent and non-human primate nervous systems. Neuron *110*, 2242–2257.e6. https://doi.org/10.1016/j.neuron.2022.05.003.

49. Xue, Y., Wang, C., Li, H., Du, S., Zhong, Y., Zhang, Y., Wang, S., Guo, K., Hou, X., Kang, D.D., et al. (2025). Lipid Nanoparticles Enhance mRNA Delivery to the Central Nervous System Upon Intrathecal Injection. Adv. Mater. *37*, e2417097. https://doi.org/10.1002/adma.202417097.

50. Gao, K., Han, H., Cranick, M.G., Zhao, S., Xu, S., Yin, B., Song, H., Hu, Y., Clarke, M.T., Wang, D., et al. (2024). Widespread Gene Editing in the Brain via In Utero Delivery of mRNA Using Acid-Degradable Lipid Nanoparticles. ACS Nano *18*, 30293–30306. https://doi.org/10.1021/acsnano.4c05169.

51. Hauck, E.S., and Hecker, J.G. (2022). Non-Viral Delivery of RNA Gene Therapy to the Central Nervous System. Pharmaceutics *14*, 165. https://doi.org/10.3390/pharmaceutics14010165.

52. Carpenter, M.D., Kus, N., Choi, R.B., Said, H., Dumitru, A.M., Bauer, J.H., Wang, S., Luks, V., Teerdhala, S., Shah, A., et al. (2025). Systemic mRNA-LNP administration in fetuses improves survival in a mouse model of spinal muscular atrophy. Mol. Ther. Methods Clin. Dev. *33*, 101599. https://doi.org/10.1016/j.omtm.2025.101599.

53. Tong, H., Ma, Z., Li, D., Yu, J., Zhu, Q., Shi, H., Zheng, Y., Zhou, L., Li, H., Lv, P., et al. (2025). Tuning the polyethylene glycol-lipid anchor length of lipid nanoparticles to enhance brain-targeted siRNA delivery. Acta Biomater. *207*, 591–602. https://doi.org/10.1016/j.actbio.2025.10.018.

54. Greene, D., Thys, C., Berry, I.R., Jarvis, J., Ortibus, E., Mumford, A.D., Freson, K., and Turro, E. (2024). Mutations in the U4 snRNA gene RNU4-2 cause one of the most prevalent monogenic neurodevelopmental disorders. Nat. Med. *30*, 2165–2169. https://doi.org/10.1038/s41591-024-03085-5.

55. Guzman, R.E., Sierra-Marquez, J., Bungert-Plümke, S., Franzen, A., and Fahlke, C. (2022). Functional Characterization of CLCN4 Variants Associated With X-Linked Intellectual Disability and Epilepsy. Front. Mol. Neurosci. *15*, 872407. https://doi.org/10.3389/fnmol.2022.872407.

56. Ali Khan, A., Valera Vazquez, G., Gustems, M., Matteoni, R., Song, F., Gormanns, P., Fessele, S., Raess, M., and Hrabě de Angelis, M.; INFRAFRONTIER Consortium (2023). INFRAFRONTIER: mouse model resources for modelling human diseases. Mamm. Genome *34*, 408–417. https://doi.org/10.1007/s00335-023-10010-7.

57. (1993). GeneReviews(®). In Seattle (WA): University of Washington, Seattle Copyright © 1993-2026, University of Washington, Seattle. GeneReviews Is a Registered Trademark of the University of Washington, Seattle, M.P. Adam, S. Bick, G.M. Mirzaa, S.E. Wallace, and A. Amemiya, eds..

58. McGraw, K.O., and Wong, S.P. (1996). Forming inferences about some intraclass correlation coefficients. Psychol. Methods *1*, 30–46. https://doi.org/10.1037/1082-989x.1.1.30.

59. Gwet, K.L. (2008). Computing inter-rater reliability and its variance in the presence of high agreement. Br. J. Math. Stat. Psychol. *61*, 29–48. https://doi.org/10.1348/000711006x126600.

60. Wongpakaran, N., Wongpakaran, T., Wedding, D., and Gwet, K.L. (2013). A comparison of Cohen's Kappa and Gwet's AC1 when calculating inter-rater reliability coefficients: a study conducted with personality disorder samples. BMC Med. Res. Methodol. *13*, 61. https://doi.org/10.1186/1471-2288-13-61.

61. Gisev, N., Bell, J.S., and Chen, T.F. (2013). Interrater agreement and interrater reliability: key concepts, approaches, and applications. Res. Soc. Adm. Pharm. *9*, 330–338. https://doi.org/10.1016/j.sapharm.2012.04.004.

62. Landis, J.R., and Koch, G.G. (1977). The measurement of observer agreement for categorical data. Biometrics *33*, 159–174.

OMTA, Volume *34*

Supplemental information

Optimized GENE TARGET framework characterizes barriers to therapeutic development for monogenic neurodevelopmental disorders

Raymond Belanger Deloge, Abigail Sveden, Zoë Frazier, Hailey Osika, Charlotte Byrne, Jillian O'Toole, Muhammed Bayraktutan, Kira A. Dies, Stephanie J. Brewster, Pilar Trelles, Morgan E. Ryan, Bo Zhang, Lisa Prock, Jed Hubbs, Elizabeth D. Buttermore, Wendy K. Chung, Audrey Thurm, Mustafa Sahin, and Maya Chopra

Table S1. Inter-rater agreement using ICC and Gwet's AC2

Category	Raters 1 to 3 (95% CI)	Raters 1 and 2 (95% CI)	Raters 1 and 3 (95% CI)	Raters 2 and 3 (95% CI)	Raters 4 and 5 (95% CI)
**Total Score***	0.67 (0.43, 0.84)	0.75 (0.47, 0.89)	0.51 (0.12, 0.77)	0.70 (0.30, 0.88)	0.32 (-0.15, 0.66)
**Criteria**					
G1°	0.86 (0.74,0.98)	0.90 (0.80,1.00)	0.78 (0.57,0.99)	0.77 (0.57,0.98)	0.59 (0.30,0.89)
E1°	0.85 (0.68,1.00)	0.76 (0.48,1.00)	1.00 (1.00, 1.00)	0.76 (0.48,1.00)	0.73 (0.54,0.92)
N °	0.95 (0.87,1.00)	0.96 (0.90,1.00)	0.94 (0.82,1.00)	0.96 (0.89,1.00)	0.89 (0.78,1.00)
E2°	0.43 (0.19,0.67)	0.37 (-0.03,0.77)	0.48 (0.20,0.76)	0.57 (0.33,0.81)	0.83 (0.68,0.98)
T1°	0.82 (0.72,0.91)	0.79 (0.68,0.91)	0.90 (0.79,1.00)	0.75 (0.60,0.90)	0.51 (0.23,0.79)
A°	0.91 (0.80,1.00)	0.93 (0.78,1.00)	0.87 (0.70,1.00)	0.94 (0.86,1.00)	0.86 (0.67,1.00)
R°	0.63 (0.50,0.76)	0.66 (0.44,0.88)	0.57 (0.42,0.72)	0.72 (0.57,0.87)	0.33 (-0.09,0.75)
G2°	0.69 (0.49,0.88)	0.77 (0.61,0.93)	0.67 (0.47,0.87)	0.65 (0.32,0.97)	0.44 (0.02,0.85)
E3°	0.63 (0.48,0.79)	0.67 (0.41,0.93)	-0.19 (-0.67,0.30)	0.63 (0.36,0.90)	0.38 (0.12,0.64)
T2°	0.80 (0.62,0.98)	0.87 (0.73,1.00)	0.56 (0.15,0.96)	0.68 (0.39,0.97)	0.52 (0.10,0.95)
**Domain**					
Clinical°	0.78 (0.67,0.89)	0.69 (0.48,0.89)	0.76 (0.58,0.95)	0.77 (0.66,0.88)	0.52 (0.18,0.86)
Genetic°	0.65 (0.48,0.82)	0.77 (0.61,0.94)	0.52 (0.17,0.86)	0.42 (0.14,0.70)	0.41 (-0.03,0.85)
Pre-Clinical°	0.77 (0.70,0.84)	0.76 (0.63,0.89)	0.77 (0.66,0.88)	0.74 (0.65,0.84)	0.48 (0.15,0.80)
Ethical°	0.64 (0.41,0.88)	0.62 (0.32,0.91)	0.21 (-0.16,0.58)	0.59 (0.21,0.96)	0.21 (-0.20,0.61)

*=Calculated using ICC
o= Calculated using Gwet's AC2

Table S2. Full GENE TARGET Scorecards for all 57 NDDs

Table S3. Criteria and Domain Scores for all 57 NDDs

Table S4. GENE TARGET Score Descriptions

**G**	0 - genetic mechanism is unknown; recommend establishing before proceeding<br>1 - genetic mechanism is unknown, but gene harbors special characteristics that can be exploited<br>2 - multi-gene CNVs account for most cases of the disorder OR conflicting or limited evidence of mechanism<br>3 - conflicting or limited evidence of mechanism, but gene harbors special characteristics that can be exploited<br>4 - loss of function or altered function of a single gene<br>6 - in addition, gene harbors special characteristics that can be exploited
**E**	0 - age of diagnosis or clinical presentation is highly variable<br>1 - diagnosis / clinical presentation in infantile to early childhood period<br>2 - features or markers typically present in prenatal imaging/testing OR clinical presentation of features at birth
**N**	0= No natural history data available; recommend natural history studies before proceeding<br>1= Cross-sectional data available<br>2= Longitudinal data available<br>3= Longitudinal data with standardized neurodevelopmental measures available
**E**	0= No validated endpoints, recommend establishing endpoints before proceeding<br>1= Neurodevelopmental endpoints (ie IQ) available<br>2= Indirect measures/biomarkers associated with neurodevelopmental phenotype available<br>3= Direct endpoints available; how patients feel, function, and survive
**T**	0 - Cell type and/or brain region not defined, no model system<br>1 - Model system under development, cell type and/or brain region not defined<br>2 - Model system available, cell type and/or brain region not defined<br>4 - Cell type and/or brain region and stage of neuronal development known and informed by a validated preclinical model, but targetability limited by properties of brain region<br>6 - Cell type and/or brain region and stage of neuronal development known and informed by a validated preclinical model, and appropriate tools are available which can access that cell type or brain region
**A**	0 - disease-modifying treatments are available and approved by regulators<br>1 - disease-modifying treatments are limited in population, symptomatic domain, or duration of use OR treatment(s) currently under evaluation

	2 - no disease-modifying treatments are available
**R**	0 - reversibility not established and resources are not available; recommend establishing before proceeding<br>1 - reversibility not established, but resources (iPSCs, animal models, organoids, etc) are publicly available and accessible<br>2 - Disorder-relevant preclinical endpoint established in a model, and ready for testing reversibility<br>3 - reversibility established but temporal window unknown<br>4 - reversibility and temporal window for rescue established
**G**	0= Gene tolerance to dosage change is unknown; recommend establishing dosage window before proceeding<br>2= Narrow therapeutic window<br>3= Wide therapeutic window suspected<br>4= Wide therapeutic window confirmed
**E**	0= Ethical principles have not been considered OR are unfavorable<br>3= Favorable risk-benefit ratio OR treatment generalizable across population<br>6= Favorable risk-benefit ratio AND treatment generalizable across population
**T**	0= Patient population not accessible or not engaged<br>2= Engaged population accessible through community organizations<br>4= Engaged population accessible through clinical cohorts or registries
